# Effectiveness of Cognitive Interventions in Older Adults: A Review

**DOI:** 10.3390/ejihpe10030063

**Published:** 2020-09-02

**Authors:** Miriam Sanjuán, Elena Navarro, M. Dolores Calero

**Affiliations:** 1 Department of Personality, Assessment and Psychological Treatment, Faculty of Psychology, University of Granada, Campus de Cartuja s/n, 18071 Granada, Spain; mirsanjuan@ugr.es; 2 Research Center on Mind, Brain and Behavior (CIMCYC), Campus de Cartuja s/n, University of Granada, 18071 Granada, Spain; mcalero@ugr.es

**Keywords:** older adults, dementia, cognitive intervention, systematic reviews, meta-analyses, aging

## Abstract

(1) Introduction: With older adults, cognitive intervention programs are most often used for preventing or reversing a decline in cognitive functions, but it has been recently noted that there are insufficient high-quality research studies that report the effects of cognitive intervention on the cognitive functioning of older adults. (2) Objective: To analyze the available evidence concerning the effect of cognitive interventions for improving or maintaining the general cognitive status of older adults who present different cognitive levels. (3) Method: a review of studies published between 2010 and 2019 using the following databases: PubMed, PsycINFO, Cochrane, Google Scholar, ProQuest and Medline. (4) Results: We selected 13 systematic reviews and/or meta-analyses. The results showed that the cognitive intervention programs improved general cognitive functioning and specific cognitive functions regardless of the initial cognitive level; that cognitive decline was slowed in older persons with dementia; and there was improvement in activities of daily living. Regarding duration of the results, benefits were maintained for periods of 2 months to 5 years. (5) Conclusion: Cognitive interventions have proven effective for maintaining and/or improving cognitive functioning in older adults regardless of their initial cognitive status. Even so, there are few studies that follow up these results to see whether they are maintained in the long term and whether there is transfer to other skills of daily life. However, we were able to observe in the present review how the participants’ cognitive level varied according to sociodemographic differences, and to identify which components of cognitive programs make them more effective. Based on the results found, we highlight the importance of designing cognitive intervention programs that meet these effectiveness criteria, in order to maximize the positive effects of such programs when working with a population of older adults.

## 1. Introduction

The aging population is an increasingly relevant topic. The percentage of older adults has continued to rise over the past decades and is expected to double in the next 25 years [1]. In 2020, 20% of the total population of the European Union (EU) is 65 or older; this proportion will rise to 30% by 2060. If the current trend continues, the rate of dependency in older adults will rise significantly across the EU over the coming decades [2].

These data anticipate an increase in the demand for professional services for elder care, given that aging is associated with an increased percentage of dependent persons. For this reason, strategies must be designed for helping older adults to remain active and independent for as long as possible.

Aging has been related to a decline in cognitive performance [3], traditionally explained from a biological perspective [4]. However, this perspective on aging as a phase of general decline was questioned by authors who noted that there are important inter- and intra-individual differences in aging [5]. In the same way, the bi-factor model of intelligence, proposed for explaining age-associated decline, has been refuted by numerous authors who have shown that a decline or impairment in skills related to fluid intelligence [6] is susceptible to reversibility through different actions taken by the individual [7].

Today, other explanations concerning cognitive decline in older adults are being considered. One idea is that cognitive decline occurs due to an environment that offers little stimulation and involvement in challenging cognitive activities [5].

According to this perspective, losses are not only due to advancing age, but often to the disuse of cognitive functions. This means that a practiced skill will be maintained over time, while a neglected skill will gradually fade and a decrease in cognitive performance will be observed [8].

This is consistent with the day-to-day reality found among older adults, where the vast majority progressively delegate activities to others, and in this way they no longer use the cognitive functions that were needed to perform them. Even though these cognitive functions may undergo certain alteration in old age, human beings have the capacity to learn [9] and therefore are able to maintain skill performance if it is practiced [10].

For cognitive decline leading to dementia, today, pharmacological therapy alone is considered to have certain clinical limitations, and its long-term therapeutic effectiveness in the cognitive realm is being questioned [11]. Non-pharmacological interventions (NPI) are shown as a viable alternative for older adults to maintain or improve their cognitive status, whether they are healthy, or present cognitive impairment [12]. Within NPI, the multidisciplinary approach is one of the fundamental principles in the interventions for older adults [13]. In this field, many scientific disciplines have developed interventions aimed to reduce dementia risk and to alleviate symptoms associated with age-related pathological processes such as cognition-oriented treatments [14], multimodal therapies [15] and Transcranial Magnetic Stimulation [16], amongst others.

Among these NPI for the treatment of cognitive decline or dementia, cognitive intervention programs are currently most used for prevention or improvement in impaired cognitive functions [17]. In fact, cognitive intervention is the NPI that has received the most empirical support whether in dementia, in normal aging or in mild cognitive impairment, leading it to be put forward as the first choice for intervention in persons with dementia [18]. Under the heading of cognitive intervention, a multitude of diverse programs are found. In order to classify this field, we will distinguish between: (1) cognitive training programs (programs that train basic cognitive strategies), (2) cognitive rehabilitation (mono or multidimensional programs on specific skills like memory, attention, arithmetic, etc.), and (3) cognitive stimulation (continuous practice programs or use of external resources).

Asserting the benefits of cognitive interventions, we find several systematic reviews. For instance, Papp et al. [19] analyzed the effects of cognitive interventions in healthy older people and concluded that the training improved immediate performance in the tasks trained, but there was no evidence of generalization to general cognitive functioning. Similarly, the review by Martin et al., [20] including a total of 36 randomized controlled trials (RCTs) between 1985 and 2007, showed that immediate memory and verbal memory improved significantly in healthy older adults after a cognitive intervention program, and also in persons with mild cognitive impairment (MCI), but the results were not generalized to their general cognitive status. Cândea et al. [21] carried out a review of 32 RCTs of the effect of cognitive intervention on working memory, both in healthy older adults and in older adults with cognitive impairment, finding improvements in the cognitive performance of subjects who had been trained in working memory, without specifying whether this improvement was generalized to overall cognitive functioning. Besides, recent reviews [14,22,23] report scientific evidence for cognitive interventions since engaging in cognitively stimulating activities can be protective for age-related cognitive decline and dementia possibly by increasing cognitive reserve and resilience in later life [14,22,23].

In the reviews cited above, cognitive intervention is found to have beneficial effects on the cognitive skills trained, but the results do not report whether these positive effects are maintained in the long term.

However, there are currently doubts about the effectiveness of cognitive interventions due to the vast heterogeneity of the studies. In 2017, the document entitled “Integrated care for older people: guidelines on community-level interventions to manage declines in intrinsic capacity,” the World Health Organization (WHO) [24] indicated that there were insufficient high-quality research studies that report the effects of cognitive intervention in the cognitive functioning of older adults, that there are imprecise estimates of the benefits and risks, and/or that the benefits are very confined and limited in relation to cost.

The objective of this review was to analyze the available evidence concerning the effect of cognitive interventions for improving and/or maintaining the general cognitive status of older adults who present different cognitive levels (healthy, with Mild Cognitive Impairment (MCI) and with dementia). The review aimed to establish the best evidence from current cognitive intervention and to identify what aspects make interventions more effective. Different types of interventions were reviewed, and their specific effects and generalization to general cognitive functioning were evaluated.

## 2. Method

We performed a bibliographic search for systematic reviews and meta-analyses of cognitive interventions in the following databases: PubMed, PsycINFO, Cochrane, Google Scholar, ProQuest and Medline. 

Scientific journals were searched for systematic reviews and/or meta-analyses between 2010 and 2019, published in English, with a European or American population, where full text was available.

Our initial search strategy included the intersection of the following terms: [“cognitive stimulation” OR “cognitive rehabilitation” OR “cognitive training” OR “cognitive therapy” OR “cognitive retraining” OR “cognitive support” OR “cognitive intervention” OR “brain training” OR “cognitive exercise” OR “memory training”] AND [“old people” OR “elderly people” OR “older adults” OR “aging” OR “cognitive ageing” OR “healthy elderly”] AND [“meta-analysis” OR “review”] NOT [“physical” OR “computer”]. 

This search was complemented by a manual search of the references from the selected reviews themselves to minimize the possibility of overlooking relevant studies.

The following inclusion criteria were used to identify study eligibility: (1) systematic reviews and/or meta-analyses that contained randomized controlled studies or clinical studies, (2) a population of healthy older adults, adults with MCI or dementia, (3) any type of cognitive intervention except computerized interventions, and (4) use of objective and/or subjective measures of results of cognitive performance. 

Studies were excluded if the main intervention was physical, or the articles did not supply data on the effect of the training in cognitive function. Computerized cognitive interventions were also excluded because the objective was to analyze tutored training, based on verbal interaction, and focused on basic, general and/or specific cognitive skills.

The present systematic review followed the recommendations and guidelines of the PRISMA guide, “Preferred Reporting Items for Systematic Reviews and Meta-Analyses” [25].

## 3. Results

### 3.1. Inclusion of Studies

**Figure 1:**
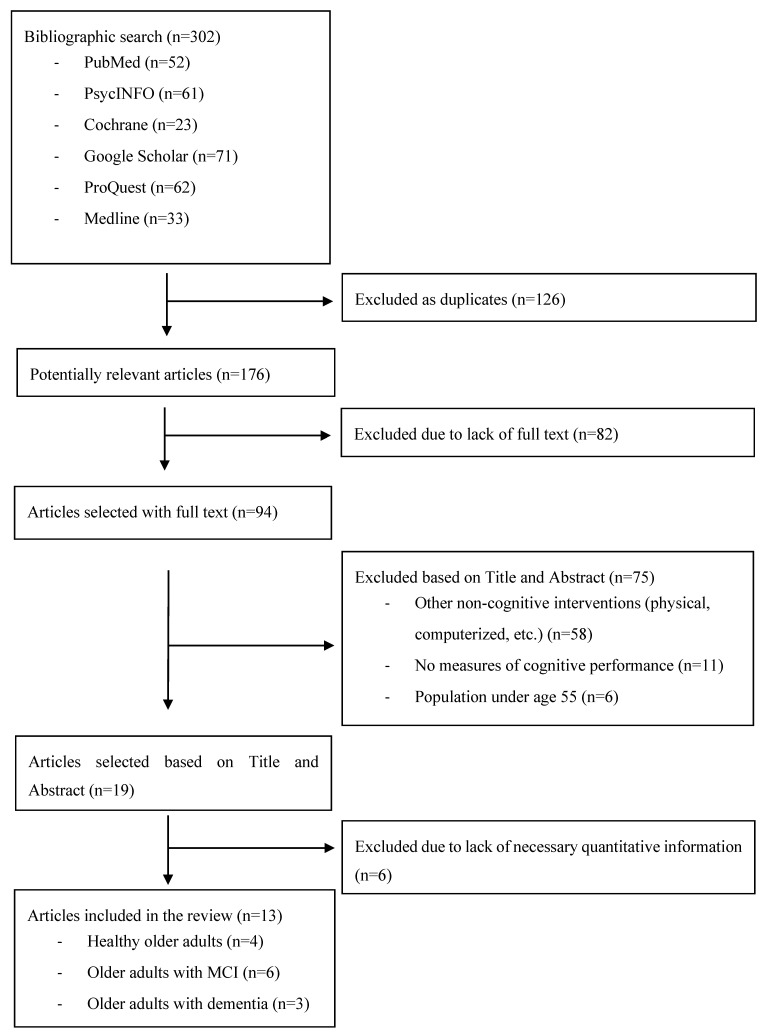
Flow chart of the literature research.

Figure 1 shows the results of the selection process. A total of 302 systematic reviews and/or meta-analyses were selected for analysis; 126 were excluded as duplicates from the different searches. Of the remaining 176, 94 were selected as containing their full text. After reviewing the abstracts, 13 articles with reviews and meta-analyses were left. Of the 13, 4 addressed a population of healthy older adults [26,27,28,29], 6 addressed a population of older persons with MCI [30,31,32,33,34,35], and 3 addressed a population of older adults with dementia [36,37,38].

The 4 reviews and meta-analyses with a population of healthy older adults included a total of 109 studies. Close analysis revealed that 27.5% of these studies were included in more than one review (30 studies were found in several reviews), so the final number of studies reviewed was 79. The 6 reviews and meta-analyses with a population of older adults with MCI included a total of 93 studies, and 41.9% of these studies were included in more than one review (39 studies were repeated), so the final number of articles reviewed was 54. Finally, the 3 reviews and meta-analyses with a population of older adults with dementia included a total of 79 studies, and 12.7% of these were included in more than one review (10 studies repeated), so in this case the final number of articles reviewed was 69. In total, 202 studies with different populations of older adults, according to their cognitive status, were analyzed.

### 3.2. Characteristics of the Participants

The following tables show the main characteristics of the studies reviewed. Table 1 contains studies with a population of healthy older adults, Table 2 includes studies with a population of older adults with MCI, and studies in Table 3 address a population of older adults with dementia.

As seen in the tables, the participants varied according to the population that the interventions were designed for. In the reviews with a population of healthy older persons (Table 1), participants were selected from the residents of the community, especially through community centers and more public locations, while in the reviews with a population of older persons with MCI (Table 2) or dementia (Table 3), participants were selected at their daycare centers, memory training clinics, and senior residences.

The sample of healthy older adults contained 9091 persons in the treatment groups and 5532 in the control groups. Only 3 reviews report the age of participants, ranging from 60 to 83 in one case [29], 60–99 in the second case [28] and 65–96 in the third [27] (mean age was between 71.8 and 72.50 years). Only one of the reviews [27] indicated the participants’ educational level, with a mean of 13.5 years of education (ranging from 9.33 [39] to 16.7 [40] years). Finally, only one of the reviews indicated gender distribution, with the male population forming 32.3% of the sample and the female population 67.7% [27].

The sample of older adults with MCI contained 2843 persons in the treatment groups and 4566 in the control groups. Only 3 reviews report the age of participants, ranging from 65 to 78 (mean age 70.3 years) [30]; 62–91 (mean age 74 years); [33] and 61–79 (mean age 73.3 years) [35]. According to two of the reviews [32,35], the mean number of years of education was 12.4, with a range from 6 [41] to 16.8 years of education [42]. According to 3 reviews [30,33,35], between 38.5% and 40% of the population was male.

Finally, the sample of older adults with dementia contained 3228 persons in the treatment groups and 830 in the control groups. The reviews report participants’ age, with ranges of 77–88 years (mean age 82.3 years) [36]; 66–87 years (mean age 76.76) [37]; and a mean age of 79.7 in another review, with no range given [38]. The mean number of years of education was 8.4 with a range from 3.7 [43] to 12.9 [44]. Regarding gender within the sample, 40% of the population was male [44].

By way of summary, it can be observed that the mean age of the participants in the group of healthy older adults and adults with MCI was quite similar. By contrast, the group of persons with dementia were older. Likewise, in the case of educational level, the groups of healthy older adults and adults with MCI are similar in this variable, while the group of persons with dementia—despite the large variability between studies—usually presents lower educational levels (mean years of education at 8.4, compared to 13.5 in the MCI group and 12.4 in the group of healthy older adults). Regarding gender, the percentage of men is around 38–40% in the three populations.

### 3.3. Characteristics of the Studies

Of the 79 studies with healthy participants, 73 (92.4%) were classified as cognitive interventions (according to 2 of the meta-analyses [26,28]: 17% general interventions, 24% on memory and attention, 27% executive function and 7% visuo-spatial. Some cases [29] included psychoeducational interventions [45] or a program based on Bandura’s theory of self-efficacy [46]). Of the 73 studies with cognitive interventions, 42 (57.5%) studies involved group interventions and 31 (42.5%) had individual interventions; 58 (79.5%) studies used passive control groups and 15 (20.5%) studies presented active control groups. Only 35 (47.9%) studies included a follow-up, from 1 month to 60 months, most of them including the follow-up study at 12 months. The interventions varied in length, averaging 10 weeks (from 2 to 24 weeks), the mean session duration was 66 min (from 45 min to 2 h) and the mean frequency was 2.5 sessions per week (range: 1 to 5 weekly sessions).

Of the 54 studies with participants with MCI, 42 (77.8%) were classified as cognitive training and 12 (22.2%) as cognitive rehabilitation. Within cognitive training we find studies using programs that worked on episodic memory [47,48], attention [49], processing speed, language, visuo-spatial skills and executive functions [50]. According to the review by Simon et al. [32], there was cognitive training that focused on memory, including the teaching of compensatory and restorative strategies, namely, errorless learning, erroneous learning, spaced recovery, visual images, associating names with faces, mental mapping, classification, hierarchical organization and the Loci method [38,47,49,51,52,53,54,55,56,57]. In addition, certain studies investigated the effects of external memory aids, such as calendars and agendas [48,51,54]. Of the total interventions, 36 (66.7%) involved group interventions and 18 (33.3%) had individual interventions. Passive control groups were used in 31 (57.4%) studies, active control groups in 15 (27.8%) studies, and 8 (14.8%) studies did not have a control group. 40 (74.1%) of these included a follow-up study, from 2 months to 28 months afterward. The intervention sessions in these studies varied in length, with a mean of 12 weeks (6 to 52 weeks of treatment); they held 1–2 sessions per week, each lasting approximately 1½ hours (Table 2).

Of the 69 studies with participants with dementia, 34 (49.3%) were classified as cognitive training, 6 (8.7%) as rehabilitation, and 29 (42%) as cognitive stimulation. There were 24 (34.8%) studies that involved group interventions and 45 (65.2%) that had individual interventions. Passive control groups were used in 33 (47.8%) studies; 22 (31.9%) studies presented active control groups and 14 (20.3%) studies did not have a control group. Only 8 (13.8%) of the studies included a follow-up study, from 1 month to 10 months afterward. The intervention sessions in these studies lasted 12.7 weeks (a range of 3 to 60 weeks of treatment), with 1–2 sessions per week of approximately 1½ hours each (Table 3).

In general, we observed that most studies applied group cognitive intervention programs in the healthy older adults and adults with MCI, while individual rehabilitation programs were applied in older adults with dementia. Most studies included control groups, but less than 50% included follow-ups. Treatment duration was usually three months in studies with healthy older adults and adults with MCI. This period was considered optimal in the specialized bibliography [28,29,30,31,32,35]. In the case of persons with dementia, the duration of the intervention was usually longer. Weekly periodicity of the sessions was more variable in the studies we examined (from 1–5 weekly sessions), so no general criterion could be established.

### 3.4. Measures of Results and Effects

All the literature we consulted included measures of results in general cognitive functioning, cognitive functioning of specific capacities (memory, attention, executive function and visuo-spatial capacity), functional capacity and emotional state.

In the studies of healthy older adults, the results include measures of memory (74.6% of the studies); attention (60.6%); executive function (52.1%); general cognitive functioning (28.2%); general emotional state (15.5%); visuospatial ability (14.9%); and functional ability (7%).

In the studies of older adults with MCI, the results include measures of general cognitive functioning (81.3%); memory (74.4% of the studies); executive function (59%); general emotional state (43.6%); attention (35.9%), functional ability (30.8%); visuospatial ability (5.1%) and quality of life (12.8%).

In the studies of older adults with dementia, the results include measures of general cognitive functioning (93.5%); general emotional state (41.3%); functional ability (39.1%); quality of life (28.3%); behavior problems (23.9%); memory (2.2%); attention (2.2%), and executive functioning (2.2%).

While in most cases there is insufficient data to determine the percentage of cases in which each instrument had been used, it was possible to observe which instruments were used the most—regardless of the group of older adults studied:

**General cognitive functioning:** the most used instrument was the MMSE (Mini-Mental State Examination) [58] (in over 70% of the studies). There are also studies that use: MoCA (Montreal Cognitive Assessment) [59], RBANS (Repeatable Battery for the Assessment of Neuropsychological Status) [60], CAPE (Clifton Assessment Procedures for Elder People) [61].

**Memory**: MMQ (Multifactorial Memory Questionnaire) [56] MFQ (Memory Functioning Questionnaire) [62], RBMT (Rivermead Behavioral Memory Test) [63].

**Attention**: CDRS-attention, TMT-A (Trail Making Test-A) [64], d2 [65], RBANS-attention [66].

**Executive function:** Reasoning test, WCST (Wisconsin Card Sorting Test) [67], Raven Matrices [68].

**Visuo-spatial capacity:** Mental rotation test, RBANS-visuospatial, LPS 50+.

**Functional capacity:** Lawton and Brody Instrumental Activities of Daily Living Scale [69], Barthel Index of Activities of Daily Living [70].

**Quality of Life:** QOLQ (Quality of Life Questionnaire [71]

**Frame of mind:** BDI (Beck Depression Inventory) [72], STAI (State-Trait Anxiety Inventory) [73], GDS (Geriatric Depression Scale) [74].

In general, we found that the constructs most often measured to assess the effects of the interventions were general cognitive functioning, memory, attention, executive function and visuo-spatial ability. The greater the cognitive decline in the sample, the greater the use of instruments that measured functional ability, quality of life and emotional state; and in groups with dementia, measures of behavioral problems were included.

### 3.5. Main Results Found in the Systematic Reviews and/or Meta-Analyses

The different reviews offered positive results from treatment with cognitive interventions for improving cognitive status in older adults. The review by Bhome et al. [26] indicates that there was a positive effect in general cognitive functioning with an effect size of 0.13 (according to their meta-analysis) and a significant improvement in psychological well-being with an effect size of 0.25, in the 5 studies from the review that evaluated well-being [26]. The review by Chui et al. [27] shows significant improvements in: (1) general cognitive functioning with an effect size of 0.42 (calculated from 41% of the studies contained in the review); (2) memory with an effect size of 0.35 (61%); (3) attention with an effect size of 0.22 (61%); (4) executive function with an effect size of 0.42 (72%); and (5) visuo-spatial ability with an effect size of 0.18 (19%). In the review by Kelly et al. [28], we found that 19 out of 26 studies (73%) presented gains in memory; 17 out of 29 (58.6%) gains in executive function; and in 9 out of 10 studies (90%) that measured transfer, there was transfer to tasks within the same domain [75,76,77,78] in 5 studies (19%), and transfer to other cognitive domains [77,79,80,81,82] in six studies (29%). Finally, the review by Reijnders et al. [29] also showed significant improvements in memory in 17 out of 21 studies (81%), executive function in 8 out of 21 (38.1%), and in general cognitive functioning and functional ability in 2 of the studies analyzed.

As for the older adults with MCI, the results of these systematic reviews and meta-analyses show that cognitive intervention can increase their general cognitive function [30,35] and specific cognitive functions such as memory [48,49,52,78,83], executive functioning [29,47,54], visuo-spatial capacity [50], attention and processing speed [83]. More specifically, we observed the following data: in the review by Smart et al. [30], a positive effect in general cognitive functioning was observed in 9 studies (100%) with an effect size of 0.37 (which varied from 0.03 [84] to 0.88 [45] according to the study). The review by Chandler et al. [31] showed significant gains in general cognitive functioning in 60% of the studies with an effect size of 0.21 analyzed from 18 of the 30 studies contained in the review. Significant gains in mood were also found in 7 out of 12 studies (58.3%), with an effect size of 0.16; in metacognition in 10 out of 20 studies (50%), with an effect size of 0.30; in functional ability in 11 out of 20 studies (55%), with an effect size of 0.23; and in quality of life in 2 out of 10 studies (20%), with an effect size of 0.10. In their review, Gates et al. [33] found gains in objective memory in 4 of 5 (80%) studies, with an effect size of 0.30, and gains in mood in 2 studies that measured change in mood produced by cognitive interventions in older adults with MCI. Finally, the review by Li et al. [35] indicated significant gains in general cognitive functioning, with an effect size of 0.41 (based on 15 of the 20 (75%) studies that the review contains); episodic memory with an effect size of 0.45 (12 out of 20 studies; 60%); semantic memory with an effect size of 0.33 (3 out of 20 studies; 15%); executive function with an effect size of 0.27 (7 out of 20 studies; 35%); visuo-spatial ability, with an effect size of 0.43 (4 out of 20 studies; 20%); attention, with an effect size of 0.35 (6 out of 20 studies; 30%); and functional ability with an effect size of 0.27 (6 out of 20 studies; 30%).

Finally, the results of the systematic reviews and meta-analyses show that cognitive intervention applied to persons with dementia can delay cognitive impairment and improve activities of daily living [38]. The review by Lobbia et al. [36] showed that 9 out of 12 studies (75%) presented significant gains in general cognitive functioning; 4 out of 9 (44.4%) presented gains in quality of life; 4 out of 8 (50%) gains in depressive symptoms, and 2 out of 6 (33.3%) gains in behavioral problems. In the study by Spector et al., [85] we found improvement in anxiety levels, and in the study by Paddick et al., [86] improvement in praxis and memory. However, in the review by Oltra-Cucarella et al. [37], improvement results are contradictory. Even so, Kurz et al., [38] support the conclusion that cognitive interventions improve general cognitive ability in older adults with dementia, finding significant data in this direction in 11 out of 18 studies (61.1%), with an effect size of 0.21.

Regarding duration and maintenance of the results, maintained benefits were verified in healthy older persons for periods of 2 months [27] to 5 years [28] after treatment. This maintenance of results was verified in 20% of the studies in the review by Bhome et al., [26]; in 35.5% in the studies reviewed by Chiu et al., [27]; in 54.2% of those reviewed by Kelly et al., [28] and in 38.1% of the studies in the Reijnders et al. review [29].

In persons with MCI, positive effects showed variable duration, although in most cases they were maintained at least 2 months after the intervention [32]. With this population, benefits were maintained over periods from 2 weeks [31] to 2 years [32]. The duration and maintenance of effects were studied in most of the reviews with older adults with MCI, with findings of long-term maintenance in 22.2% of the studies from Smart et al. [30], 54.2% of the studies from Chandler et al. [31], 30% of the studies from Gates et al. [33], and 35% of the studies from Li et al. [35], with effect sizes of 0.33 in general cognitive functioning; 0.25 in executive function; 0.50 in attention; 0.65 in quality of life; 0.10 in functional ability and 0.38 in mood [35].

Finally, in the older adults with dementia, the data are inconclusive, given that two [36,37] of the three reviews did not report duration of effects or follow-on data. Even so, Kurz et al. [38] found in 3 of their 7 studies (42.9%), where follow-up was evaluated, that benefits were maintained beyond the treatment period in cognitive ability [21,83] and in memory performance [49].

In short, we observed a significant improvement in general cognitive functioning, memory, attention, executive function, visuo-spatial ability and functional ability, regardless of the group of older adults in question. Moreover, in healthy adults, benefits from the cognitive interventions were seen in psychological well-being as well as in transfer to other tasks. There were also gains in mood and metacognition in older adults with MCI, and gains in anxiety, praxis, depressive symptoms and behavioral problems in older adults with dementia. Moreover, both the older adults with MCI and those with dementia improved their quality of life after a cognitive intervention.

In all groups, the effects of the training persisted for at least the 2 months following. Nevertheless, long-term follow-up must be included in order to verify these effects [39,81].

## 4. Discussion

The principal objective of the present review was to examine the effectiveness of cognitive interventions designed for older adults. We sought to analyze the aspects or variables that make them more effective, for the ultimate purpose of encouraging cognitive program designs that produce the most benefits in a population of older adults.

After cognitive intervention, all the systematic reviews and meta-analyses obtained positive results in the specific skills that were trained. These effects were observed to offer benefits not only in specific trained skills, but also in general cognitive function and in transfer to emotional well-being and quality of life. These results concur with prior reviews by Papp et al. [19], Martin et al. [20] and Cândea et al. [21].

When analyzing the effectiveness of cognitive intervention programs in older adults, several aspects were taken into account: what type of population was targeted, what types of skills were being trained, how the training was structured and the way that its effectiveness was evaluated. Mean age of the samples was also taken into account, as well as gender, mean level of education, and place of residence. There were differences in mean age in the studies with a healthy older population, with MCI and with dementia (71.6, 72.6 and 79.4, respectively). There were also variations in the percentage of male population (32.3%, 38.5% and 40%, respectively) and the mean years of education received (13.5, 12.4 and 8.4, respectively). These data concur with results reported in prior reviews that groups with greater impairment are older in age and have lower education levels [87]. There were also differences between the samples in relation to where they had been selected. The healthy older adults lived in their homes and were selected at community and civic centers, while the other two groups were selected at day care centers, memory training clinics and senior residences.

Cognitive training was the intervention used most often in all populations (92.4% in healthy older adults, 77.8% in adults with MCI and 49.3% in adults with dementia). When there was greater impairment in a sample, another cognitive treatment was used, such as cognitive rehabilitation in older adults with MCI (22.2%) or with dementia (8.7%), and cognitive stimulation in the latter population (42%).

As for the mode of application, carrying out cognitive intervention programs in group settings seems to have additional benefits for cognitive performance [28]. In this regard, we observed that group settings were the format of choice in the studies analyzed with healthy older adults (57.5%) or with MCI (66.7%). These results are supported by research showing that cognitive interventions produce maximum benefits when participants train in groups [88]. This may be because the group setting offers an opportunity for participants to support each other [88]; it may increase motivation [89] and allow people to share their concerns and feelings about their cognitive problems [90]. However, with more advanced cognitive impairment comes increasing use of individual treatment. In older adults with dementia, only 34.8% of the studies used a cognitive intervention in a group format.

This would be an important consideration when organizing and designing such programs: we gather from the foregoing analysis that implementation of group programs is the most adequate for healthy older adults and adults with MCI, while individual interventions are a more adequate option in older adults with dementia.

Regarding the number of sessions needed or the duration needed for the cognitive intervention to be effective, the data are somewhat contradictory [35]. What emerges from the data in the different reviews is that most studies recommended 10–12 weeks of treatment. In healthy persons, a 10-week duration was the average [26,27,28,29], and with increasing cognitive impairment, the number of sessions for applying cognitive treatment increased (about 12.7 weeks in older adults with dementia) [36,37,38]. Regarding session length, we also find that the average length increases with greater cognitive impairment. Sessions lengthen from the 66 min average session in healthy older adults [26,27,28,29] to 92 min in older adults with MCI [30,31,32,33,34,35] and 90 min in older adults with dementia [36,37,38].

Another critical concern is that studies should include follow-up, in order to learn whether benefits found in the cognitive interventions are maintained over time. Unfortunately, we found the studies to be variable in this aspect and not all of them conducted follow-up. There are variations according to the type of population, such that in persons with MCI, follow-up was implemented in 74.1% of the cases; while in healthy older adults, this drops to 47.9% and in the dementia population only 13.8% included follow-up.

Regarding the type of measures, we observed that, regardless of cognitive status, assessment addressed memory, attention, executive function, general cognitive ability, functional ability and mood, to a greater or lesser degree. Studies with healthy older adults were the ones that most used measures of memory (74.6%), attention (60.6%) and processing speed (14.9%). Studies of older adults with MCI were the ones that most used measures of executive function (59%) and mood (43.6%) and studies of older adults with dementia made the most use of measures of general cognitive functioning (93.5%), functional ability (39.1%) and quality of life (28.3%). In this regard, we observed that with greater cognitive impairment, other noncognitive factors take on greater importance in the older adult’s ability to carry on with daily living and have quality of life.

Most of the studies showed that a cognitive intervention produced improvement in general cognitive functioning, whether in healthy older adults (63.1% of the studies), with effect sizes from 0.13 to 0.42; in older adults with MCI (67.5% of the studies), with effect sizes from 0.37 to 0.41; or in older adults with dementia (68.1% of the studies), with effect sizes of 0.21.

Aside from general cognitive functioning, we also found benefits in healthy older adults in specific variables such as memory (with an effect size of 0.35); attention (with an effect size of 0.35); executive function (with an effect size of 0.42); visuo-spatial ability (with an effect size of 0.18) and psychological well-being (with an effect size of 0.25), in 68.9%, 64.8%, 55.9%, 19.4% and 25% of the studies, respectively. In the case of older adults with MCI, we found gains in memory (with an effect size between 0.30 and 0.45); attention (with an effect size of 0.35); executive function (with an effect size of 0.27); visuo-spatial ability (with an effect size of 0.43); mood (no effect size data); metacognition (with an effect size of 0.30); functional ability (with an effect size between 0.23 and 0.27) and quality of life (with an effect size of 0.10), in 51.7% 30%, 35%, 20%, 31.8%, 50%, 42.5% and 20% of the studies, respectively. Finally, in older adults with dementia, we found improvement in quality of life, depressive symptoms and behavioral problems (in 44.4%, 50% and 33.3% of the studies, respectively, with effect size data not reported).

Furthermore, the efficacy and effects of the cognitive intervention on older adults’ cognitive functioning were also shown to increase when other components such as decreasing stress and anxiety, participation in challenging, novel cognitive tasks, social participation, physical activity and healthy sleep habits were included, as can be observed in 7 of the 20 studies (35%) in the review by Bhome et al. [26]. In addition, personal/internal strategies (like using mnemonic rules) and environmental/external strategies (like using calendars, agendas, etc.) improved or maintained cognitive performance [91] according to 3 of the studies [48,51,54] found in the review by Simon et al. [32].

As for long-term maintenance of benefits offered by cognitive interventions, this was studied in 37%, 35.4% and 42.9% of the studies with healthy older adults, adults with MCI and adults with dementia, respectively. In all these studies, we find that the effects of training can be retained for at least two months, whether in memory or in executive domains, for healthy older adults and for adults with MCI [29,92]. According to contributions from Kelly et al. [28], it is possible to maintain these effects over a longer term if maintenance strategies are added [90,93], with reinforcement sessions or an adaptive training paradigm [39,80,94], with at least ten intervention sessions [39,81].

Finally, one of the most interesting results included in these reviews is the transfer of benefits from cognitive interventions to the adult’s other cognitive domains or other functional abilities. In this regard, 3 of the reviews report transfer data: (1) the review by Kelly et al. [28] which includes specific data on transfer in healthy older adults; (2) the review by Stott et al. [34] with data on transfer in older adults with MCI, and (3) the review by Kurz el at. [38], in older adults with dementia. Kelly et al. [28] include studies (66.6%) of healthy older adults that show how there was transfer of the cognitive intervention effects to tasks within the same cognitive domain (16.7% of the studies that contained transfer data) [19,87,95]. They also indicated that transfer had been produced to untrained cognitive domains (25% of the studies analyzed) [39,79,80,82,96,97]. In this case, they reported that transfer depended on the type and duration of the training [88,89,90]. Specifically, interventions that used adaptive, repetitive sessions [71,77,82,98] or longer periods of training [39,82,96] showed effects of transfer to daily life. Even so, we find that 74.4% of the studies do not include measures of transfer to daily life [29,99], so we are not able to determine the impact of cognitive interventions at this level. There were also several studies in the review by Reijnder et al. [29] that specifically addressed the question of generalization to untrained tasks, or to the subjective experience of cognitive functioning [45,76,80,82,96,98], but did not offer data on transfer to the older adult’s everyday life or to their functional ability. In this regard, we may say, along with Kelly et al. [28], that there is very little evidence of transfer effects to situations of daily life in healthy older adults. For this reason, the question of whether the effects of cognitive interventions are generalized to improvement in activities of daily living must be addressed more explicitly in future research. Furthermore, the topic of transfer is only addressed very generally in two reviews of older adults with MCI and with dementia [34,38], so it is not possible to draw conclusions for these populations.

In short, based on this review, we have found that cognitive interventions produce maintenance and/or improvement of the skills trained, thereby having an impact on the cognitive ability of older adults regardless of their initial cognitive level. This improvement is seen in overall cognitive capacity and in specific cognitive abilities such as memory, attention, executive functions, etc. Moreover, certain authors [100] have corroborated that cognitive interventions lead to changes in basic cognitive functions, with these changes possibly being applied to real-life situations, due to an increase in their functional skills as well.

Summarizing our findings, we highlight the following program aspects as particularly important in making programs more effective:

A minimum of 10 weeks of treatment with two sessions per week [28].

- Session length of 60 to 90 min [26,27,28,29,30,31,32,33,34,35,36,37,38].
- Cognitive interventions implemented in a group format in healthy adults and adults with MCI [5,26].
- Study follow-ups included [26,27,28,29,30,31,32,33,34,35,36,37,38].
- Several cognitive skills worked on at the same time [101].
- Inclusion of other components related to quality of life, such as decreasing anxiety and stress; participation in challenging activities and novel cognitive tasks; social participation; physical activity; and healthy sleep habits [26].
- Employment of personal or internal strategies (like using mnemonic rules) and environmental or external strategies (using calendars, agendas, etc.) [32].
- Measures of daily functioning included, in order to analyze whether the improvement in cognitive functioning is generalized to activities of daily living.

However, the present review is limited by the characteristics of the studies found that were reviewed. In this regard it must be noted that methodological rigor was lacking in the studies included in these reviews: there was a lack of primary results, sample sizes were generally small and therefore not representative, and the methodologies applied were quite diverse. The interventions were heterogeneous (different intervention formats, different durations, some included an active control group and others a passive one, etc.), and most contained multiple components, so the interventions were classified according to their most prominent domain, leading to the possibility that the most effective component of the intervention may not have been adequately detected. Attempting to address the previous limitations, we considered it important to carry out randomized controlled trials (RCTs) using high quality methodology, given that on some occasions the lack of such well-designed RCTs gives rise to a negative opinion of cognitive intervention programs.

## 5. Conclusions

To date, as we have observed throughout this study, cognitive interventions are effective in older adults regardless of their initial cognitive status.

In conclusion, our review endorses the effectiveness of cognitive interventions, and highlights the aspects that make them more effective. Across Europe, with its aging population and the implied social and health challenges, there is a need for strategies that aim to keep older adults active and independent as long as possible. This review shows that the design of cognitive interventions that meet the effectiveness criteria stated above can help to improve cognitive functioning and quality of life for older adults. We believe, therefore, that European policies that aim to foster successful aging must consider this type of scientific review when implementing cognitive programs in an older population, in order to achieve maximum effectiveness.

## Figures and Tables

**Table 1:** Systematic reviews and meta-analyses addressing a healthy older population.

Review	Main Characteristics	Participants	Interventions	Skills Measured	Follow-On	Effect Size
Bhome et al., (2018)	Comm. Context 22 RCTs No patients with MCI or dementia, or significant psychiatric or physical comorbidities	Total N: 1639 **TG** = 824 **CG** = 815 **MA** = no data **GEN** = no data **YE** = no data **CF** = no data	**TG** = Cognitive training (by applying manual or self-applied strategies) **CG** = Passive control, waiting list, informational talks or group activities at the community center **FORMAT** = Group interventions **FREQUENCY** = Programs from 4 to 16 weeks, especially 8-week programs, with approximately 1.5 h sessions, from 1 to 3 weekly sessions)	General cognitive functioning Subjective psychological well-being	All pre-post 4 follow-ups (between 1 and 6 months).	0.13 General cognitive functioning 0.23 Subjective psychological well-being
Chiu et al., 2017	Comm. Context 31 RCTs Interventions in cognitive training Older adults with normal cognitive functions, with no diagnosis of MCI or dementia	N total = 5499 **TG** = 3555 and **CG** = 1944 **MA** = 72.57 (from 65 to 96) **GEN** = 67.7% (F); 32.3% (M). **YE** = 13.5 **CF** = 26.8	**TG** = cognitive training (of the 31 RCTs, 14 analyzed general cognitive function, 20 memory, 20 attention, 22 executive function and 6 visuo-spatial capacity) **CG** = Most were passive control groups, 5 studies with a waiting list and 5 studies with informational talks or some type of education. **FORMAT**: 13 studies with group interventions and 17 studies with individual interventions **FREQUENCY**: Session duration approx. 1 h (20 min to 2 h). From 1 to 5 weekly sessions, from 2 to 20 weeks, and from 8 to 60 total sessions according to the study.	General cognitive functioning Memory Attention Executive function Visuo-spatial capacity	All pre-post 10 follow-ups (2, 3, 4, 6 months, 1 or 2 years depending on the study)	0.42 General cognitive functioning 0.35 Memory 0.22 Attention 0.42 Executive function 0.18 Visuo-spatial capacity
Kelly et al., 2014	Comm. Context 31 RCTs > 10 participants per condition Intervention of cognitive training or general mental stimulation Participants: > 60 years without CI	Total N = 4555 **TG** = 2192 and **CG** = 2363 **MA** = 71.8 (from 60 to 99) **GEN** = no data **YE** = no data **CF** = no data	**TG** = cognitive training and interventions with mental stimulation **CG** = Passive and active (DVD or educational lectures, training in health promotion, computer games without mental training, or some type of non-structured learning). **FORMAT** = 7 studies with group interventions and 24 studies with individual interventions **FREQUENCY** = Session duration approx. 1 h (30 min to 2 h). From 1 to 5 weekly sessions, from 2 to 24 weeks, and from 8 to 75 total sessions according to the study.	General cognitive functioning Memory Executive function	All pre-post 16 follow-ups (2, 3, 4, 6, 8 or 9 months, 1, 2, 3 or 5 years depending on the study).	No data available
Reijnders et al., 2013	Comm. Context and clinical settings 27 RCTs - 21 Healthy older adults - 6 E. with MCI 8 Clinical Studies Participants: healthy older adults or with MCI Any type of cognitive intervention Subjective and/or objective measures of results	N totalhea = 2520 N totalMCI = 410 **TGhealthy** = 1499 **CGhealthy** = 1021 **TGMCI** = 216 **CGMCI** = 194 **MAhealthy** = 71.6 (from 60 to 83) **MAMCI** = 69.4 (from 60 to 78) **GEN** = no data **YE** = no data **CF** = no data	**CT** = cognitive interventions (memory, executive function, attention, etc.) **CG** = 10 studies with healthy participants and 2 with MCI had passive control groups (waiting list) and 11 studies with healthy participants and 4 with MCI had active control groups. **FORMAT**: Healthy: Group interventions in 10 studies, individual interventions in 10 studies and 1 study used both MCI: Group interventions in 4 studies and individual interventions in 2 studies **FREQUENCY**: Healthy: Session duration approx. 1 h (30 min to 2 h) From 3 to 180 weeks. MCI: Session duration approx. 1 h (30 min to 2 h) From 5 to 20 weeks.	**Healthy/MCI** 21/6 RCTs: Memory 8/2 RCTs: Executive function 3/0 RCTs: Intelligence (fluid) 2/0 RCTs: Attention 1/3 RCTs: General cognitive functioning 2/1 RCTs: Functional capacity	All studies are **pre-post**. **Healthy**: 5 studies included follow-on data from one year later. **MCI**: 2 studies included follow-on data from 3-4 months later.	No data available

Note. TG = treatment group; CG = control group; MA = mean age; GEN = gender; YE = mean years of education; CF = cognitive function; MCI = mild cognitive impairment.

**Table 2:** Systematic reviews and meta-analyses addressing an older population with mild cognitive impairment (MCI).

Review	Main Charac-Teristics	Participants	Interventions	Skills Measured	Follow-On	Effect Size
Smart et al., 2017	Clinical context 9 RCTs Cognitive interventions participants >60 years old with MCI diagnosis	N total = 676 **TG** = 378 **CG** = 298 **MA** = 70.31 (from 65 to 78) **GEN** = 72.6% (F); 27.4% (M). **YE** = no data **CF** = no data	TG = traditional cognitive training CG = 2 studies with passive control groups (waiting list) and 7 studies with an active control group. **FORMAT**: All were group interventions except one study with individual training. **FREQUENCY**: Session duration approx. 1.5 h (45 min to 2.5 h). From 1 to 3 weekly sessions, from 4 to 24 weeks, and from 6 to 72 total sessions according to the study.	General cognitive functioning Functional capacity	All **pre-post** 7 short-term follow-ups (2 weeks to 3 months)	0.38 General cognitive functioning
Chandler et al., 2016	Clinical context 24 RCTs Cognitive interventions Participants with MCI	T total N = 1100 C total N = 783 **TG** = 1100 **CG** = 783 **MA** = no data **GEN** = no data **YE** = no data **CF** = no data	**TG** = 24 Cognitive interventions **CG** = 14 studies with passive control groups (waiting list) and 10 studies used active control groups. **FORMAT**: 21 group interventions and 3 studies with individual training. **FREQUENCY**: Session duration approx. 2 h. From 1 to 3 weekly sessions, from 4 to 48 weeks, and from 4 to 78 total sessions according to the study.	General cognitive functioning Functional capacity Frame of mind Quality of life	All **pre-post** 8 studies with therapist-based interventions (from 1 to 28 months) 5 multimodal studies (from 1 to 18 months)	0.21 General cognitive functioning 0.23 Functional capacity 0.16 Frame of mind 0.10 Quality of life
Simon et al., 2012	Clinical context 14 RCTs 6 studies without a control group, of which 1 is a single case study. Cognitive intervention Participants with MCI	Total N = 3575 **TG** = 580 **CG** = 2995 **MA** = no data **GEN** = no data **YE** = 11.86 **CF** = no data	**TG** = cognitive training (especially in episodic memory) **CG** = 6 studies without a control group. 11 studies with passive control groups (4 studies with a waiting list) and 3 studies with active control groups: informational talks or some type of education. **FORMAT**: 10 studies with group interventions and 10 studies with individual interventions **FREQUENCY**: Session duration approx. 1.5 h (45 min to 2 h). From 1 to 5 weekly sessions, from 2 to 12 weeks, and from 6 to 30 total sessions according to the study.	Memory Functional capacity Frame of mind	All **pre-post** 10 follow-ups (2, 3, 6 months, 1 or 2 years depending on the study).	No data available
Gates et al., 2011	Clinical context 5 RCTs, 2 UCTs (uncontrolled population) 3 NRCTs (non-randomized population) Cognitive intervention Participants with MCI	Total N = 305 **TG** = 169 **CG** = 136 **MA** = 74 (from 62 to 91) **GEN** = 57.84% (F); 42.16% (M). **YE** = no data **CF** = 26.3	**TG** = 6 studies with cognitive training (3 RCTs, 2 UCTs, 1 NRCT) and 4 studies with training in memory strategies (2 RCTs, 2 NRCTs). **CG** = 2 studies without a control group, 5 studies with passive control groups (2 studies with a waiting list) and 3 studies with active control groups **FORMAT**: 4 studies with group interventions and 2 studies with individual interventions and 4 studies with no information on format **FREQUENCY**: 1 h session duration (45 min to 2 h). From 1 to 5 weekly sessions, from 3 to 52 weeks, and from 6 to 100 total sessions according to the study.	General cognitive functioning Memory Attention Executive function Visuo-spatial capacity Functional capacity Frame of mind	All **pre-post** 4 follow-ups (3, 5 and 6 months depending on the study).	0.30 Memory
Stott and Spector 2011	Comm. Context and clinical settings 3 RCTs, 3 UCTs (uncontrolled population) 2 NRCTs (non-randomized population) Cognitive intervention, cognitive rehabilitation or memory interventions. Participants with MCI	Total N = 280 **TG** = 172 **CG** = 108 **MA** = no data **GEN** = no data **YE** = no data **CF** = no data	**TG** = 7 studies with cognitive training and 3 studies with cognitive rehabilitation **CG** = 2 studies without a control group and 8 studies with passive control groups (2 studies with a waiting list) **FORMAT**: 4 studies with group interventions and 6 studies with individual interventions **FREQUENCY**: 2 h session duration (45 min to 2 h). From 1 to 5 weekly sessions, from 3 to 12 weeks, and from 3 to 30 total sessions according to the study.	General cognitive functioning Memory Functional capacity Frame of mind	All **pre-post** No follow-up data	No data available
Li et al., 2011	Clinical context 12 RCTs 8 studies without a control group Stimulation/ Cognitive training or Cognitive Rehabilitation. Participants with MCI	Total N = 690 **TG** = 444 **CG** = 246 **MA** = 73.3 (from 61 to 79) **GEN** = 54.2% (F); 45.8% (M) **YE** = 12.9 **CF** = no data	**TG** = Cognitive training or Cognitive Rehabilitation. **CG** = 8 studies without a control group, 7 studies with passive control groups (6 studies with a waiting list) and 5 studies with active control groups **FORMAT**: 9 studies with group interventions and 11 studies with individual interventions **FREQUENCY**: Session duration 1.5 h (45 min to 2 h). From 1 to 5 weekly sessions, from 2 to 14 weeks, and from 5 to 103 total sessions according to the study.	General cognitive functioning Memory Executive function Visuo-spatial capacity Frame of mind	All **pre-post** No follow-up data	0.41 General cognitive functioning 0.45 Memory 0.35 Attention 0.27 Executive function 0.43 Visuo-spatial capacity 0.35 Frame of mind 0.27 Functional capacity 0.32 Quality of life

Note. TG = treatment group; CG = control group; MA = mean age; GEN = gender; YE = mean years of education; CF = cognitive function; RCT = randomized controlled trial; UCT = uncontrolled trial; NRCT = non-randomized controlled trial.

**Table 3:** Systematic reviews and meta-analyses addressing an older population with dementia.

Review	Main Characteristics	Participants	Interventions	Skills Measured	Follow-On	Effect Size
Lobbia et al., 2018	Clinical context 12 RCTs Dementia diagnosis MMSE score >10 Cognitive stimulation intervention	Total N = 873 **TG** = 505 **CG** = 368 **MA** = 82.3 (from 77 to 88) **GEN** = 67.5% (F); 32.5% (M). **YE** = no data **CF** = no data	**TG** = Cognitive stimulation **CG** = 3 studies without a control group, 1 study with passive control group and 8 studies with active control groups: entertainment activities, crafts, music, etc. **FORMAT**: All interventions were individual. **FREQUENCY**: From 1 to 2 weekly sessions, from 7 to 14 weeks, and 14 total sessions.	General cognitive functioning Specific cognitive functioning (language, memory, attention, executive function, praxis and orientation) Quality of life Symptoms of depression and anxiety Communication capacity	All **pre-post** No follow-up data	No data available
Oltra-Cucarella et al., 2018	Clinical context 33 RCTs Dementia diagnosis Cognitive intervention	Total N = 1240 **TG** = 778 **CG** = 462 **MA** = 76.16 (from 66 to 87) **GEN** = 71.4% (F); 38.6% (M). **YE** = 8.7 **CF** = no data	**TG** = 20 studies with cognitive training, 5 with cognitive stimulation, 2 with cognitive rehabilitation and 6 studies that combine cognitive training and cognitive stimulation. **CG** = 24 studies with a passive control group, 1 study with an active control group (relaxation and psycho-education) and 8 studies without a control group. **FORMAT**: All are individual interventions. **FREQUENCY**: From 4 to 60 weeks and from 5 to 120 total sessions according to the study (mean = 32.2 sessions).	General cognitive functioning Memory Attention Executive function Functional capacity Quality of life	All **pre-post** No follow-up data	No data available
Kurz et al., 2011	Adult Daycare Centers (CS) Memory clinics or research centers (CT and CR) 33 RCTs Dementia diagnosis Cognitive intervention	Total N = no data **TG** = 1945 **TG**(CS)= 1361 **TG**(CT/CR) = 584 **CG** = no data **MA** = 79.7 **GEN** = 51% (F); 49% (M). **YE** = 8 **CF** = 18.8	**TG** = 20 Cognitive stimulation (CS) studies and 13 Cognitive Training (CT) or Cognitive Rehabilitation (CR) studies. **CG** = 13 studies with a passive control group and 20 with an active control group **FORMAT** (CS): 9 group interventions and 11 studies with individual training. **FREQUENCY** (CS): From 4 to 56 weeks and from 6 to 103 total sessions according to the study. **FORMAT** (CS/CR): 11 group interventions and 3 studies with individual training. **FREQUENCY** (CS/CR): From 3 to 24 weeks and from 5 to 60 total sessions according to the study.	General cognitive functioning Functional capacity Frame of mind Quality of life	All **pre-post** 8 follow-ups (from 1 to 10 months)	0.21 General cognitive functioning

Note. TG = treatment group; CG = control group; MA = mean age; GEN = gender; YE = mean years of education; CF = cognitive function; CS = Cognitive Stimulation; CT = Cognitive Training; CR = Cognitive Rehabilitation.
